# Cognitive Function in Individuals with Chronic Hypoparathyroidism—A Prospective Observational Study

**DOI:** 10.1210/clinem/dgae800

**Published:** 2024-11-15

**Authors:** Adelina Tmava-Berisha, Astrid Fahrleitner-Pammer, Tatjana Stross, Simon Geiger, Christina Geiger, Frederike Fellendorf, Mario Scherkl, Alexander Finner, Anna Holl, Nina Dalkner, Eva Reininghaus, Karin Amrein

**Affiliations:** Department of Psychiatry and Psychotherapeutic Medicine, Medical University of Graz, 8036 Graz, Austria; Division of Endocrinology and Diabetology, Department of Internal Medicine, Medical University of Graz, 8036 Graz, Austria; Department of Psychiatry and Psychotherapeutic Medicine, Medical University of Graz, 8036 Graz, Austria; Division of Endocrinology and Diabetology, Department of Internal Medicine, Medical University of Graz, 8036 Graz, Austria; Division of Infectiology, Department of Internal Medicine, Medical University of Graz, 8036 Graz, Austria; Department of Psychiatry and Psychotherapeutic Medicine, Medical University of Graz, 8036 Graz, Austria; Division of Pediatric Radiology, Department of Radiology, Medical University of Graz, 8036 Graz, Austria; Department of Psychiatry and Psychotherapeutic Medicine, Medical University of Graz, 8036 Graz, Austria; Department of Psychiatry and Psychotherapeutic Medicine, Medical University of Graz, 8036 Graz, Austria; Department of Psychiatry and Psychotherapeutic Medicine, Medical University of Graz, 8036 Graz, Austria; Department of Psychiatry and Psychotherapeutic Medicine, Medical University of Graz, 8036 Graz, Austria; Division of Endocrinology and Diabetology, Department of Internal Medicine, Medical University of Graz, 8036 Graz, Austria

**Keywords:** hypoparathyroidism, cognitive function, parathormone, neuropsychological test battery

## Abstract

**Objective:**

“Brain fog” is a frequently reported, distressing experience among individuals with chronic hypoparathyroidism, characterized by reduced concentration and reduced ability to perform day-to-day tasks. However, evidence linking chronic hypoparathyroidism to cognitive impairment is limited and inconsistent. This study aimed to explore cognitive function in these patients using a validated neurocognitive test battery, compare results with a matched healthy control group, and analyze the frequency of cognitive impairment based on normative data.

**Methods:**

The participants’ cognitive performance was tested using a cognitive test battery, including the Trail Making Test A/B, the Color-Word Interference Test, and the California Verbal Learning Test. These tests were used to evaluate the cognitive domains of attention and processing speed, verbal learning and memory, and executive function. In total, 30 individuals with hypoparathyroidism and 30 healthy controls were included.

**Results:**

Twenty-four patients were women (80.0%), with a median age of 44.5 ± 13.1 and a median disease duration of 8.7 years (±5.3). Individuals with chronic hypoparathyroidism showed poorer cognitive performance in attention and processing speed [*F*(1,57) = 8.65, *P* = .005*, *η*^2^ = 0.13] compared to healthy controls. A significantly higher percentage of patients had cognitive deficits in both attention and processing speed (56.7% vs 3.3%) and executive function (60.0% vs 16.7%).

**Conclusion:**

This study provides evidence that cognitive dysfunction, particularly in attention and processing speed, is common in chronic hypoparathyroidism. Recognizing cognitive impairment in these patients is crucial, especially when discussing workability. Neuropsychological training as an adjunct therapy strategy may be beneficial in managing these cognitive deficits.

Significance Statement This study reveals that cognitive dysfunction, particularly in attention and processing speed, is common in chronic hypoparathyroidism, a previously underexplored area. Using a comprehensive neurocognitive test battery, we found a significantly poorer cognitive performance in patients compared to matched healthy controls. These findings highlight the importance of recognizing cognitive impairment in clinical management and discussions about patients’ workability. We suggest incorporating neuropsychological training as a potential adjunct therapy to manage cognitive deficits, offering a novel approach to improving the quality of life for individuals with this rare but devastating endocrine disease.

Hypoparathyroidism (HypoPT) is a rare endocrine disorder characterized by low or absent PTH levels with consecutive hypocalcemia and hyperphosphatemia (1). This condition predominantly affects females and is caused mainly by the accidental removal or injury of the parathyroid glands during neck surgery (2). Depending on the course of the disease, it can be transient, recovering in the first months after surgery, or permanent (chronic) when it lasts more than 6 months (3). PTH is important in maintaining calcium homeostasis through the reabsorption of calcium and excretion of phosphate in the kidney, regulation of bone turnover, and vitamin D-mediated gastrointestinal absorption of Ca2+ (2, 4). The clinical manifestations include a wide range of signs and symptoms such as paresthesia, tetany, muscle cramps, nephrolithiasis, cardiac alterations, and neuropsychological symptoms, all associated with decreased quality of life (3, 5-7). A recent retrospective study analyzing the medical history of patients with chronic HypoPT showed that the comorbidities associated with HypoPT play a key role in hospitalizations and premature mortality (8). Supplementation of calcium and vitamin D analogs represents the basis of conventional therapy, although this does not restore calcium homeostasis completely (3, 9). The recent approval of the recombinant human PTH (1-84) and trans-con PTH are fundamental advances in this direction; however, due to the high cost, there is a need for the strict selection of patients (3).

Cognition is the mental process of obtaining knowledge and comprehension through thinking, experiences, and perception (10). The term “cognitive deficit” describes the impairment of various cognitive domains and is not specific to any particular disease or condition. Instead, it can manifest an individual's underlying health issue (11). Cognitive dysfunction in HypoPT has been a topic of interest in the last few decades; however, the recent evidence is limited and primarily based on case reports, as summarized in a recent review by Sardella et al (12). Although the underlying mechanism is still not clear, intracranial calcification and hypocalcemia have been associated with cognitive dysfunction, including decreased attention, memory, processing speed, and extrapyramidal symptoms (13-16). Moreover, there is evidence regarding the direct effect of PTH and the expression of the PTH2 receptor (PTH2R) in the brain, suggested to participate in the limbic, neuroendocrine, and sensory protecting functions (17). PTH can influence the regional cerebral capillary blood flow and modulate the calcium and phosphate homeostasis in the brain, playing an important role in brain cell functionality (18). Noteworthy, the pathological changes in serum PTH levels could potentially result in neuropsychological effects also through PTH2R, as highlighted by Usdin et al (19).

“Brain fog” is a common and distressing experience for individuals with HypoPT, characterized by symptoms affecting the central nervous system, such as confusion, reduced concentration, and impaired cognitive function (20). These symptoms are associated with a reduced quality of life and impaired work ability and, consequently, high utilization of social security benefits in individuals with HypoPT (21, 22). To date, cognitive changes in HypoPT have primarily been documented through patients’ self-assessments of well-being rather than objectively evaluating the cognitive function (23-25).

This study addresses existing research gaps by examining the cognitive function of individuals with chronic HypoPT. We hypothesized that individuals with chronic HypoPT have worse cognitive performance than a control group. Furthermore, we anticipate a higher frequency of cognitive impairment in HypoPT patients than in controls, compared to normative data, emphasizing the clinical relevance of our research.

## Materials and Methods

### Participants

Individuals with chronic HypoPT treated at the Clinical Department of Endocrinology and Diabetology of the Medical University Graz, Austria, were invited to participate in the study. This investigation was part of the ongoing study “HypoPT-the building of a prospective cohort-29-062 ex 16/17,” which aims to identify and examine patients with HypoPT in Austria and to create a registry of this cohort. Patients were diagnosed using the criteria of the European Society of Endocrinology publications on the management of adult chronic HypoPT (26). The inclusion criteria were diagnosis of chronic HypoPT (regardless of etiology), age between 18 and 70 years, and IQ > 80. Patients with neurological (for example, Parkinson's or Alzheimer's) or psychiatric diseases (for instance, major depressive episode, mania, or schizophrenia), as well as insufficient German language knowledge, were excluded from the study. Regarding metabolic and cardiovascular diseases, all participants underwent a comprehensive medical history review to identify any significant comorbidities. The participants were recruited during the timeframe spanning from 2016 to 2021. For healthy controls (HC), we used the data of the HC of the ongoing study “BIPLONG-25-335 ex 12/13,” which used the same cognitive tests. Concerning HC, persons with a psychiatric disorder, medical history of brain injuries or thyroid/parathyroid disorders, and assumption of psychoactive drugs were excluded from the study. From the BIPLONG data set (n = 748), a group of 30 HC was matched for age, sex, and education to the patient group of n = 30. All participants provided written informed consent before participation in the study. The Medical University of Graz Ethics Committee approved the study following the Declaration of Helsinki (29-062 ex 16/17).

### Neuropsychological Assessment

The cognitive performance of the participant was assessed using a cognitive test battery that included the Trail Making Test (TMT) Part A/B (27), the Color-Word Interference Test by J.R. Stroop (28), and the California Verbal Learning Test (CVLT) (29). All tests were provided in German. The raw scores were summed up to 3 cognitive domain scores: attention and processing speed, verbal learning and memory, and executive function. For this purpose, the TMT-A and the Color and Word part of the Stroop test were combined into the domain score “attention and processing speed.” The TMT-A measures individual attention and psychomotor speed, while the Color and Word part of the Stroop measures selective attention, visual processing, and cognitive flexibility. The subscales of the CVLT were summed up into the domain score “verbal learning and memory.” The Stroop interference test measures response inhibition and response selection, indices of executive control, while the TMT-B measures cognitive flexibility and executive functioning. Both were included in the domain score “executive function.” Additionally, the IQ was assessed with the Multiple-Choice Vocabulary Intelligence Test (30). Self-reported depressive symptoms were tested by the German version of the Beck Depression Inventory-II (BDI-II) (31). A trained investigator administered the cognitive test battery.

### Statistics

We compared the demographic variables of patients with chronic HypoPT and HC using *t*-tests for continuous variables and chi-square tests for categorical ones. For nonnormally distributed continuous variables, we utilized the Mann–Whitney *U* test. Regarding the cognitive raw scores, it is important to note that higher TMT A/B scores and Stroop scores reflect worse performance, and therefore scores were inverted. Then, Z-transformation was performed to sum up cognitive raw scores to domain scores, resulting in higher scores reflecting higher performance in all domains. Since the normal distribution was not given for the domain scores, a Rankit transformation of these scores was performed. Three univariate analyses of variance were calculated to test for differences between cognitive domain scores between patients and HC. In addition, according to the test manuals, chi-square tests were performed to compare the frequency of cognitive deficits between patients and HC considering the age-based norm reference values (32-34). A cognitive deficit was defined as a score below the 25th percentile. Based on this, we calculated a deficit score (yes/no) for each domain. A memory deficit was defined as a deficit in 3 out of 5 CVLT subtests (below the 25th percentile). An attention deficit was defined as a score below the 25th percentile in at least 2 of 3 attention tasks (TMT A, Stroop Word Reading, Stroop Color Reading), and a deficit in executive function was defined as a deficit below the 25th percentile in 1 of the 2 executive function tasks (TMT B, Stroop interference). Error probabilities below .05 were accepted to denote statistical significance. IBM SPSS version 28 was used to perform data analyses.

## Results

### Descriptive Statistics

A total of 60 individuals were included in the statistical analyses; 30 had a diagnosis of chronic HypoPT and 30 were matched HC (see Table 1). Furthermore, no significant differences were observed between the groups regarding their IQ. The etiology of chronic HypoPT was predominantly postsurgical, accounting for 90% of patients. The remaining 10% were classified as either idiopathic or autoimmune. The median average disease duration at the evaluation was 8.8 years (±5.2). The majority of HypoPT patients were treated with conventional therapy (80%), and a small group (20%) was on Natpara therapy. For further details, please see Table 1. According to the BDI-II, patients with HypoPT had a significantly higher score than the HC group. However, the mean value of BDI-II (11.5 ± 8.4) was well below the cut-off of a clinically relevant depression (BDI-II >18 points).

**Table 1.** Baseline demographic and clinical variables for study participants

	Patients (n = 30)	Healthy controls (n = 30)	Statistics
Sex n (%)			
Women	24 (80.0)	25 (83.3)	*χ^2^* (1) = .111; *P* = .739
Men	6 (20.0)	5 (16.7%	
Age, mean ± SD	44.5 ± 13.1	44.0 ± 12.8	*t*(58) = 0.038; *P* = .882
Education, years, mean ± SD	13.6 ± 2.2	13.4 ± 2.2	*t*(55) = 0.450; *P* = .655
BDI-II, mean ± SD, points	11.5 ± 8.4	4.5 ± 5.2	*t*(48.3) = 4.054*; P* < .001*
IQ, mean ± SD, points	105.3 ± 13.5	112.3 ± 15.5	*t*(56) = −1.840; *P* = .071
Clinical characteristics			
Duration of disease (years)	8.7 ± 5.3		
Etiology			
Postsurgical	27 (90.0)		
Idiopathic	2 (6.7)		
Autoimmune	1 (3.3)		
Treatment			
Standard therapy	24 (80)		
PTH 1-84	6 (20)		

Data are given mean ± SD or n (%).

Abbreviations: BDI, Beck Depression Inventory.

^*^Significance set at *P* < .05.

### Neuropsychological Test Performance

Table 2 presents the differences between individuals with HypoPT and HC in cognitive raw scores. HypoPT patients had significantly lower cognitive test performance in almost all cognitive raw scores.

**Table 2.** Cognitive raw scores in patients and controls

Cognitive variables, M ± SD	Patients (n = 30)	Healthy controls (n = 30)	Test statistic (*F*)	*P*-value	*η* ^2^
TMT-A (s)	35.6 ± 16.9	28.1 ± 8.8	4.625	.036*	0.074
TMT-B (s)	80.6 ± 38.4	66.88 ± 18.7	3.085	.084	0.050
TMT-B- TMT-A (s)	47.4 ± 34.6	38.8 ± 14.4	1.581	.214	0.068
Stroop color-word reading (s)	46.4 ± 10.9	46.1 ± 10.4	0.012	.912	0.000
Stroop color naming (s)	70.0 ± 27.1	32.9 ± 9.6	49.920	**<**.001*	0.463
Stroop interference (s)	108.6 ± 38.8	73.1 ± 14.4	21.999	**<**.001*	0.275
CVLT trial 1-5	48.4 ± 15.4	59.3 ± 9.1	11.188	.001*	0.162
CVLT short delay free recall	10.0 ± 3.9	12.3 ± 3.1	6.087	.017*	0.095
CVLT short delay cued recall	11.5 ± 3.3	13.2 ± 2.4	5.369	.024*	0.085
CVLT long delay free recall	10.8 ± 3.4	12.8 ± 2.6	6.528	.013*	0.101
CVLT long delay cued recall	11.9 ± 3.4	13.5 ± 2.5	4.243	.044*	0.068
CVLT recognition	14.2 ± 1.4	14.8 ± 0.6	4.374	.041*	0.070

Abbreviations: CVLT, California Verbal Learning Test; M, mean; s, seconds, TMT, Trail Making Test.

^*^Significance set at *P* < .05.

Considering the significant difference of the BDI-II between the groups, we did an analysis of covariance comparing patients with HC in the cognitive domains of attention and processing speed, verbal learning and memory, and executive function by controlling the effect of BDI-II as a covariate. The univariate results showed that groups (HypoPT vs HC) differed in the attention and processing speed domain, while no difference was found in the other cognitive domains (see Table 3).

**Table 3.** Univariate results following the 1-way analysis of covariance

	Patients (n = 30)		Healthy controls (n = 30)				
Cognitive domain scores	M	SD	M	SD	F	*P*-value	*η* ^2^
Attention and processing speed	−0.90	2.54	0.90	1.52	8.64	.005*	0.132
Verbal learning and memory	−1.83	5.87	1.83	3.95	2.04	.159	0.035
Executive function	−9.04	3.22	0.91	2.61	1.512	.224	0.026

^*^Significance set at *P* < .05.

Abbreviations: M, mean.

In addition, chi-square tests were performed to calculate the frequency of cognitive deficits of patients and controls, comparing the cognitive performance of both groups with the age-based norm reference percentile values. As shown in Table 4, among 30 patients with HypoPT, 17 out of 30 (56.7%) had a deficit in attention and processing speed, 14 of them (46.7%) had a deficit in verbal learning and memory, and 18 out of 30 (60.0%) had a deficit in executive function. In addition, the chi-square test showed a significant difference between patients and HC in the frequency of cognitive deficits in attention and processing speed [*χ^2^*(1) = 20.317, *P* < .001] and executive function [*χ^2^*(1) = 11.915, *P* < .001]. There was no significant difference between groups in the verbal learning and memory domain [*χ^2^*(1) = 3.590, *P* = .058].

**Table 4.** Cognitive deficits of patients and controls, comparing with the age-based norm reference values

Deficit domain	Patients (n = 30)	HCs (n = 30)	Statistics
Attention and processing speed deficit (%)	17 (56.7)	1 (3.3)	*χ^2^(1)* = 20.317, *P* < .001*
Verbal learning and memory deficit (%)	14 (46.7)	7 (23.3)	*χ^2^(1)* = 3.590, *P* = .058
Executive function deficit (%)	18 (60.0)	5 (16.7)	*χ^2^(1)* = 11.915, *P* < .001*

Abbreviations: HC, healthy controls.

^*^Significance set at *P* < .05.

## Discussion

In this study, we found that individuals with chronic HypoPT performed worse in the attention and processing speed domain compared to the HC group, while no significant difference between the groups was found in verbal learning and memory and executive function. Our analysis showed a higher frequency of cognitive impairment in attention and processing speed and executive function in patients compared to controls.

Although the literature on this topic is limited and mainly based on isolated case reports or series of patients and nonspecific, self-administered questionnaires (16, 35, 36), our results support the association between HypoPT and objectively measurable cognitive dysfunction, as demonstrated in a recent systematic review (12). In accordance with our study, a pilot cross-sectional study (n = 19) conducted in the United States (37) showed that impaired cognition was common in HypoPT, especially slower processing speed. We additionally used a control group, a larger sample size, and a standardized neuropsychological battery, supporting and expanding their findings. Additionally, Saponaro et al (38) found that patients with chronic HypoPT following thyroid surgery did not exhibit severe cognitive impairment. However, their study also indicated that specific cognitive functions, including visuospatial attention, executive function, and semantic memory, are influenced by serum calcium levels, with a significant correlation observed between low albumin-corrected calcium levels and poorer cognitive performance. These findings underscore the importance of monitoring and managing serum calcium levels to prevent subtle cognitive impairments. It is worth noting that Saponaro et al's study involved patients with a shorter disease duration (6.5 years) compared to our study (8.8 years), which might explain some differences in outcomes. Additionally, the severity of the disease in their cohort may differ from that in our study.

On a biochemical level, it is also important to consider the direct role of calcium associated with the PTH2R in the brain. It is known that calcium allows astrocytes to support information processing (39). Such information integration may be disrupted in patients with hypocalcemia due to HypoPT. This mechanism has been supported by Rubin et al (37), who found a correlation between slower processing speed and lower serum calcium levels. HypoPT can cause extensive intracranial calcification, including the white matter of the parietal and frontal lobes (40), possibly affecting, among others, the speed performance of individuals, assumed from the already known positive relationship of processing speed with the white matter volume (41). A recent study from Denmark (42) combined assessments of quality of life, cognitive function, biochemistry, and magnetic resonance imaging-based head imaging to explore the possible pathogenesis of “brain fog” in HypoPT. The results showed that patients with HypoPT reported a lower quality of life compared to controls, as measured by general questionnaires and disease-specific patients’ reported outcome tools. Objective measurements of cognitive function using neuropsychological tests revealed that patients performed worse than controls, particularly in long-term memory, a domain of the hippocampus. In support of this, magnetic resonance imaging analysis showed that the hippocampus was smaller in patients with HypoPT, and smaller size was linked to more severe cognitive impairment. In addition, the duration of the disease was linked to the volume of the thalamus in individuals who had undergone surgery. The duration of the disease appears to be a determining factor in cognitive dysfunction, which may explain the differences in results between Skiajer's study and ours. Furthermore, correlations were observed between the volumes of specific brain substructures and distinct neurocognitive functions in individuals with HypoPT. Additional associations were identified between the volumes of certain brain regions and particular cognitive functions in patients with HypoPT, such as thalamus volume and auditory memory, caudate volume and executive functions, and left putamen volume and processing speed. This preliminary investigation indicates a potential link between the cognitive deficits experienced by patients with HypoPT and alterations in brain structure. It highlights the need for additional research into the topic. In addition, an alternative potential explanation for cognitive dysfunction in HypoPT refers to hyperphosphatemia associated with basal ganglia calcification progression (13). A growing body of evidence suggests that cognition could also be directly affected by disruptions of direct PTH action (43). It has been suggested that dysfunctional brain PTH2Rs and their specific natural agonist, tuber infundibular peptide of 39 residues may play a role in nociceptive information processing in the spinal cord, the regulation of various psychotropic neurons in the hypothalamus, and the modulation of emotional processes (44). Furthermore, the lack of interaction of PTH with the joint PTH2R-tuber infundibular peptide of 39 residues could also be at least partly responsible for the cognitive dysfunction in HypoPT (44). Exploring the neurobiological impact of PTH and the impact of PTH replacement therapy on patients’ cognitive function could bring valuable insights into this topic.

Furthermore, the epidemiological evidence of the inverse relationship between depression and cognitive dysfunction (45, 46) has to be considered. Findings from previous studies demonstrated an increased risk of depression in HypoPT, using primarily well-being assessments, such as the Symptom Checklist-90-Revised or 36-item Short Form Health Survey, a self-report of impaired quality of life (5, 23). Rubin et al (37) explored the relationship between cognitive abilities and emotional function among hypoparathyroid adults using the National Institutes of Health Toolbox Emotion Battery. They found no correlation between cognitive and emotional function, including sadness, whereby they did not use a standard test for depression. Being aware of a possible confounder effect of depression on the results, we included the BDI-II, a standardized instrument for the severity of depression, as a control variable in our analysis. Although not the main focus of this work, the significant difference between patients and controls in the BDI-II scores is noticeable and should be further explored, supplemented by clinical interviews and the clinician-administered scales used in diagnosing depression. Furthermore, our results demonstrate a higher frequency of cognitive impairment in HypoPT patients in the cognitive domains of executive function and attention and processing speed, emphasizing the clinical relevance of the topic. Executive function includes working memory, mental flexibility, inhibition, and impulse control—skills that enable people to follow directions, focus, control emotions, and attain goals (47). In addition, attention and processing speed are clinically relevant cognitive measures that play an important role in everyday activities (41, 48, 49). Based on the quantifiable cognitive deficits observed in HypoPT patients, our study supports a possible link between cognitive dysfunction and health-related poor quality of life among HypoPT patients, including physical health problems and worse social functioning (21, 50, 51). Exploratory research examining alterations in brain structure over the course of the illness would provide valuable insights into the topic.

A strength of our study is that we used a standardized neuropsychological test battery that covers a wide range of cognitive abilities, such as attention and processing speed, verbal learning and memory, and executive function, which are essential for optimal daily function and provide a good overview of an individual's cognitive skills. In addition, the patient's data were compared to a parathyroid control group and normative data. The study's limitations include that our cohort is relatively small and homogenous—primarily women with predominantly postsurgical etiology, reflecting the typical HypoPT cohort. In addition, it is limited by the lack of precise information regarding the dosage and duration of the ongoing therapy. Furthermore, a correlation analysis between test results and neuroimaging features could have provided additional insights into the relationships between cognitive outcomes and underlying neural mechanisms. The absence of essential biochemical parameters poses another constraint on the study, rendering it impossible to analyze correlations between these parameters and the participants’ cognitive performance. Cognitive function is a long-term parameter; therefore, 1 serum calcium measurement performed during neuropsychological testing should be interpreted cautiously. In addition, recent evidence shows cognitive symptoms can occur in the context of a “calcium crash” despite “normal” blood calcium levels. A random calcium level does not capture daily calcium fluctuation (43). A follow-up study design exploring the cognitive performance of HypoPT patients over time, considering the possible variability of the biochemical parameter (inclusive at-home calcium monitoring device) and looking at functional magnetic resonance imaging, would provide valuable insights and is currently being performed in Denmark.

In conclusion, this study provides evidence that cognitive dysfunction is a prevalent feature of chronic HypoPT. Recognizing the presence of cognitive impairment in patients with HypoPT is crucial in clinical settings to assess work ability, tailor interventions, adjust treatment strategies, and enhance overall patient care, ultimately improving quality of life and functional outcomes. Therefore, we suggest incorporating neuropsychological training into the treatment program of HypoPT patients as an integral component of comprehensive care to improve cognitive function and associated coping strategies for daily challenges. It is unknown if modern, more physiologic treatment options improve these aspects.

## Data Availability

Some or all datasets generated during and/or analyzed during the current study are not publicly available but are available from the corresponding author on reasonable request.
